# The Defining Role of Environmental Self-Identity among Consumption Values and Behavioral Intention to Consume Organic Food

**DOI:** 10.3390/ijerph16071106

**Published:** 2019-03-28

**Authors:** Haroon Qasim, Liang Yan, Rui Guo, Amer Saeed, Badar Nadeem Ashraf

**Affiliations:** 1 School of Economics and Management, China University of Geosciences (Wuhan), Wuhan 430074, China; ruiguo@cug.edu.cn (R.G.); badarfcma@gmail.com (B.N.A.); 2 School of Business and Economics, University of Management and Technology, Lahore 54770, Pakistan; amer.saeed@umt.edu.pk; 3 School of Economics and Management, East China Jiao Tong University, Nanchang 330013, China

**Keywords:** organic food, consumption values, environmental self-identity, behavioral intention

## Abstract

Consumption values and self-identity are the essential antecedents of consumer sustainable behavior. By integrating the theory of consumption values and self-identity approach, this research explores the relationship among consumption values (functional, social, conditional, epistemic and emotional), environmental self-identity and the behavioral intention to consume organic food. The data was collected from 406 organic food consumers through a structured questionnaire in Lahore (Pakistan). Using the PLS-SEM approach, we find that conditional value, emotional value, epistemic value, and functional value quality have a significant positive influence on consumers’ behavioral intention to consume organic food. We further find that environmental self-identity significantly mediates the structural relationship between consumption values and the behavioral intention to consume organic food. Our results imply that the interventions targeting environmental self-identity are a promising way to promote sustainable consumption behavior. Our findings also have important implications for the development of the organic food market based on consumption values and self-identities.

## 1. Introduction

The industrial revolutions and expeditious economic development, especially over the last two centuries, have led to immense over-consumption and deterioration of natural resources. As a result, nowadays the world is facing severe environmental challenges such as climate change, air and water pollution, deforestation and soil degradation [1]. Due to threatening environmental conditions and ecological imbalances, consumers are getting more vigilant for the consequences which their consumption habits might have for the environment [2]. Recently, the organic food market has witnessed a significant expanding trend due to an upsurge of consumer environmental concerns [3]. Numerous studies have mentioned the willingness of consumers to pay a premium price for organic features [4]. This has encouraged firms to invest in green products, by instigating key innovations and alterations in the production processes [3,5]. Consumers’ organic food choice has vital importance for firms and food policymakers to attain sustainable consumption and development.

Recent research has considered different aspects of consumer behavior for green products [6]. For instance, some studies report that the characteristics of green products (e.g., less pollution, the economy of resources and recycling potential) might evoke consumer emotions to protect the environment and influence their consumption behavior [7]. Similarly, situational factors, such as environmental problems or incentives on green products, also encourage people to go green [8]. Likewise, social surroundings [9] and peer opinions [10] also influence consumers’ green purchase decisions. Consumer knowledge has also been reported as a factor which has a significant impact on consumer decision making process [11,12]. Consumers’ urge to seek novelty and to gain substantial information regarding product utility in terms of price and quality can also influence consumers’ decisions to buy organic products [13]. As a sum, this strand of the literature suggests that the primary stimulus behind consumers’ decisions to purchase green products is consumption values which consumers experience during the product usage. 

A recent parallel strand of the literature has considered the role of self-identities in sustainable consumption behaviors of individuals [14]. Consistent with self-perception theories, self-identity theory states that “a person acts on his own and others’ expectation of him [15]”. Self-identity that an individual carries might have a significant influence on his environmental preferences, intentions, and behavior [14,16]. For example, consumers generally consume a product that is compatible with their perceived identity [17]. Recently, consumer self-identities have been thoroughly examined in marketing and consumer research, such as brand association or product related identities [18,19] and consumption motivations [20]. Arguably consumption values and self-identities can significantly impact pro-environmental preferences, intentions, as well as behavior [21,22,23,24]. However, these relationships in the domain of organic food products are poorly understood. For instance, how do consumer motivations and their self-identities reflect a consistent and spontaneous connection with their organic food behavior? In this paper, we study the mediating role of environmental self-identity between the relationship of consumption values and conusmers’ organic food behavior intention. 

Past studies, examined the role of specific self-identities to determine consumer organic food behavior [25,26]. In similar, the mediating role of consumers’ organic food identity also examined between the relationship of consumers’ motivations and their behavior to consume organic food [27]. However, the result found that organic food identity did not mediate the relationship between individuals’ motivations and their organic food behavior. Our study is different from past studies, in that we analyze whether environmental self-identity mediate the relationship between consumers’ consumption values and the behavior intention to consume organic food. In this regard, some recent studies argue that consumers might have more general kind of self-identities that can be useful to predict a wide range of pro-environmental preferences, intentions and behavior for which specific self-identities are less predictive [22,28]. Therefore, different from previous research [25,27] we consider a more general type of environmental self-identity which might have a significant mediating role in the relationship between consumption values and consumer organic food behavior. 

From a marketing point of view, marketers and product designers need to know what consumer require in organic product and how interventions targeting individual environmental self-identity can promote pro-environmental behavior. Numerous studies have been conducted on the consumer’s pro-environmental behavior in Western countries [29,30,31]. However, fewer studies have obliged research on environmental aspects in Asian counterparts, including Pakistan [32,33]. Similarly, Biswas and Roy [34] call for additional research exertions to examine the progress of consumer’s attitude, intention, and behavior towards sustainable consumption in developing countries.

During the last decade, environmental problems have become more visible in Pakistan. According to the global climate risk index 2018 [35], Pakistan is one of the most affected countries due to extreme environmental conditions, which has affected the lives of many in the country. From 1997 to 2006 alone, more than 524,000 people are estimated to have died directly from extreme environmental events [35]. Similarly, adverse effects on people’s health have been witnessed due to air and water pollution. In the last five years, Punjab Province has been heavily affected by smog. According to the data revealed by the Pakistan Air Quality Initiative (PAQI), Lahore (the capital of Punjab Province) has a PAQI index value of 183 that indicates its air to be the unhealthiest [36]. Due to worsening environmental conditions, people are getting more concerned about health and environmental issues. The government and NGOs in Pakistan have also launched numerous initiatives to counter environmental problems and create pro-environment awareness among the masses. Additionally, the adverse effects of chemical fertilizer usage on people’s health have made them more conscious about their food consumption habits, which has resulted in a shifting trend from conventional food products to organic food products in Pakistan [37].

According to the statistics issued by the Research Institute of Organic Agriculture & IFOAM Organics International 2018 [38], although most of the developed nations, including Switzerland, Denmark, and Sweden, are at the top of the list of organic food per capita consumption countries, however 86% of the world’s total organic food producers are in developing nations. The same report states that 40% of the world’s organic food producers are from Asian countries and the organic agricultural land in Asia grew at a rate of 23.5% in 2016.

Pakistan’s organic food market is growing at a decent pace, making the study of organic food highly relevant to Pakistan. Organic farming is not a newly introduced concept in South Asia, as this practice has been followed for centuries in the Indo-Pak region. Traditionally with a low population, Pakistan’s agriculture was almost entirely organic and has been referred to as Desi (indigenous) or Khalis (pure) [32]. However, in the early 1960s, the usage of pesticides, fertilizer, and high-yielding varieties (HYVs) in farming practices shifted organic production to non-organic production to meet the demand for increased food production [39]. In recent years, organic food farming has gained much popularity, as the consumers’ need for nutritious and healthy food is rising in developing countries [40]. In Pakistan, since 2013 initial steps have been taken by stakeholders for the promotion of local organic food markets. Khalis farmers markets and Haryali market were pioneering organic food markets established to provide a platform for organic food producers [41]. Organic food producers can sell their products directly through these platforms to expand their product sales. The recent popularity of organic products in Pakistan has resulted in the launch of many organic food producers such as Zacky Farms, Ndoz Greens, Dali, and Reef’s Organic Box which indicates the growing trend of organic food demand in Pakistan [42]. 

Food policymakers are also taking the necessary steps to encourage organic farming in Pakistan. The National Institute of Organic Agriculture (NIOA) supports the installation of organic herbicide, organic fertilizer, and organic pesticide manufacturing units which is considered to be a key milestone in organic farming [33]. Pakistan’s organic agricultural landshare also increased from 34,209 to 45,299 hectares in 2016 [38]. It is estimated that 33% of the farmers are producing organic food in Pakistan, and the ratio is expected to double in the next few years as consumer’s demand for organic food is increased. Moreover, the annual organic product exports of Pakistan are worth about $100 million [33].

By integrating the theory of consumption values [43], and self-identity approach to sustainable behavior [44], this study aims to find the relation between consumption values and consumers’ behavior intention to consume organic food with an intervening role of general environmental self-identity between consumption values and behavior intention in the context of a developing country. Section 2 presents the theoretical context and hypotheses development. The methodology adopted to conduct this research is shown in Section 3, which leads to the presentation of empirical analysis and results in Section 4. Section 5 consists of a detailed discussion of the results while Section 6 concludes the study by elaborating conclusions, limitations and some future research directions.

## 2. Theoretical Context and Hypotheses Development

### 2.1. Conceptual Model

The literature suggests that individuals’ identities can mediate the relationship between their motivations and behavior. This can be articulated by a hierarchical motivational-identity-behavior perspective [45]. Consumer behavior models [46,47] also illustrate that identity can intervene in the relationship among consumer psychographic and behavior [48]. Belk [17] argued that consumers usually prefer products that meet their perceived identity. Past studies have examined the influence of consumption values on consumers’ sustainable behavior [6,8]. Similarly, environmental self-identity also acknowledged to significantly mediate the link between biospheric values and pro-environmental intentions and behavior [49]. Some recent studies have focused on the interrelationships of consumer motivations, self-identities and sustainable behavior [27,50,51].

**Figure 1:**
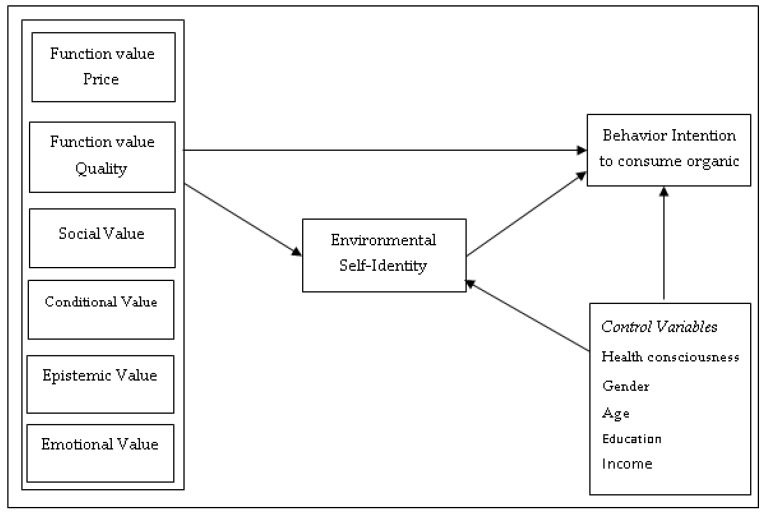
Conceptual framework.

Building on this literature, we aim to find the influencing factors of consumer behavior intention towards organic food consumption by integrating the components of consumption values theory and environmental self-identity. We conceptualize that consumption values (functional, social, conditional, epistemic and emotional) indirectly influence behavioral intention to consume organic food through environmental self-identity [25,27,50]. The proposed conceptual model is shown in Figure 1.

### 2.2. Behavioral Intention to Consume Organic Food

Following previous studies [27,51], we emphasize on behavioral intention to consume organic food rather than the actual organic food consumption behavior. Behavioral intention to consume organic products refers to the individual’s intent to purchase or consume organic products in the future [52]. Behavioral intention is considered to narrate values and identity more clearly than actual past behavior, as market factors, for example, market distance and product availability are more likely to affect actual past behavior [27,53]. Several recent studies have used the concept of behavioral intention to study the consumers’ intent to use hedonic artifacts or to adopt green products [54,55]. 

### 2.3. The Theory of Consumption Values

Values play a crucial role in decision-making and are illustrated as broad psychological constructs that could affect consumer’s interest, attitude and behavior [56]. Values are considered to be the ultimate basis of choice selection that drives buying behavior [57]. “Consumers’ overall assessment of the product’s utility based on the perception of what is received and what is given” is known as the perceived value [58]. Perceived value is the difference of give and take, where the benefits which consumer attain from the product are referred as “take,” while “give” narrates what consumer have sacrificed to obtain the product with its benefits [54]. 

The theoretical underpinning of our hypotheses is based on the theory of consumption values [43] where values are the intrinsic and extrinsic factors associated with the components of purchase [6]. It emphasizes on three facets of consumer choice behavior: (1) consumer choice of what to buy and what not to buy, (2) consumer choice of preferring one type of product to another and (3) consumer selection between different brands [43].” The theory identifies consumer choice behavior being influenced by five consumption values: functional value, social value, emotional value, epistemic value, and conditional value. These values determine the nature and direction of the relationship between the consumer and product, once the consumer derived values after interacting and experiencing product or service [59]. Lin and Huang [8], Suki [60] and Gonçalves, Lourenço and Silva [23] applied the theory of consumption values and found that consumption values influence consumer pro-environmental behavior.

#### 2.3.1. Functional Value

Functional value is considered to be a prime driver of consumer choice of organic products. It is defined as consumers’ perception of product performance based on its durability, reliability, dependability, and price [43]. It is more of the intrinsic value of the product rather than extrinsic values such as reputation or status gain with the product association. Based on its’ attributes, the functional value is categorized into two dimensions; price, and quality [13]. Consumers evaluate both the price and quality of an organic product simultaneously while making a purchase decision [7]. A consumer may prefer an organic product due to the specific attributes such as the use of natural ingredients, organic nature and health benefits [12]. Moreover, consumers also evaluate organic product performance in term of its ability to deliver economic value. If the price paid for organic product is justified in term of benefits derived from the product, then it results in a willingness to pay a premium price of organic product [61]. Therefore, both the price and quality have critical importance in consumers’ purchase decision of organic product. From this discussion, the following hypotheses are developed:

**H1-a:**  Functional value (price) has a significant positive effect on behavioral intention to consume the organic product.

**H1-b:**  Functional value (quality) has a significant positive effect on behavioral intention to consume the organic product.

#### 2.3.2. Social Value

Social pressure or peer reviews are critical driving forces behind consumer choice behavior [7,9]. Social value is defined as a perceived utility that a product/service provides due to its affiliation with one or more specific social, cultural, and socioeconomic groups [43]. It relates to self-image, especially when a product association is seen to improve consumers’ social-status [13]. In the context of organic products, social value is a perceived net utility gained from organic product consumption based on the perception regarding social pressure or status gain. Social value has a significant positive impact on sustainable consumption behavior [34]. Thus, we draw the following hypothesis:

**H2:**  Social value has a significant positive effect on behavioral intention to consume the organic product.

#### 2.3.3. Conditional Value

Conditional value is defined as the perceived net utility that an individual attains under specific circumstances or situations [43]. It arises when product usage is strongly associated with specific situations [62]. These specific situations may include the availability of subsidies or discounts on organic products, easy and nearby availability of organic products and the purchase of the organic product under worsening environmental conditions [34]. Consumer research recognizes that change in situational variables can have a significant impact on behavioral intention [63]. Conditional value has a significant positive influence on consumer pro-environmental behavior [8,64]. Therefore, we propose the following hypothesis:

**H3:**  Conditional value has a significant positive effect on behavioral intention to consume the organic product.

#### 2.3.4. Epistemic Value

Epistemic value measures the perceived net utility that consumer derives from the product’s ability to increase curiosity, provide novelty, and/or satisfying knowledge needs [43]. Existing research suggests that knowledge is a key factor in the decision making process [3]. Consumers’ desire for product novelty, compatibility and disclosure of information regarding product attributes can significantly influence their behavioral intention [65,66]. And the insufficiency of essential product information may lead to an attitude-behavior gap [67]. In the case of green product, eco-labels or third party certification may also provide substantial information to fulfill consumer desire for product knowledge. A significant positive influence of epistemic value has been reported on consumer green product purchase behavior [23]. From this discussion, we draw the following hypothesis:

**H4:**  Epistemic value has a significant positive effect on behavioral intention to consume the organic product.

#### 2.3.5. Emotional Value

Emotional value is distinguished from other constructs, as it contains not only utilitarian components but also hedonistic components [13]. It is defined as “the perceived net utility that is derived from the product’s ability to arise feeling or affective states” [43]. Product usage is mostly related to consumer emotional response. Experiences and emotions related to past product consumption help to predict future consumption outcomes of individuals with strong emotional value [68]. Receptive and enjoyable green purchase decisions result from the positive emotions associated with the product [69]. Previous research reports that emotional value has a positive impact on consumers’ choice of green products [8,23]. Thus, the following hypothesis is developed:

**H5:**  Emotional value has a significant positive impact on behavioral intention to consume the organic product.

### 2.4. Environmental Self-Identity

Identity exploration is highly relevant in determining pro-environmental behavior [51]. Numerous studies suggested the significant importance of self-identity to predict environmental preferences, intention, and behavior [22,26]. According to self-congruity consideration [70], and self-perception theories [71], individuals act in accordance with their own, and others’ expectation of them [15]. Self-identity referred to as a composition of collected roles that a person fulfilled, which in turn persuades a constant action for the endorsement of self-concept [72]. It is a label that one used to carry with him/herself which relates to a specific behavior [73]. Hence environmental self-identity is defined as “an individual’s perception of themselves as a kind of person who acts in an environmentally friendly manner” [49]. Here the conceptualization of environmental self-identity differs from environmental identity, where the latter refers to the extent to which an individual perceives that environmentalism is an essential part of who he is [44]. An individual with a higher level of environmental self-identity has a stronger belief to be a pro-environmental person and eventually more likely to act in an environment friendly way.

Previous research on environmental psychology has examined the role of specific self-identities in predicting the behavior that is consistent with those identities. For example, energy-saving self-identity can predict intentions to save energy [74], self-identity as a recycler leads to the actions causing recycling [75] and environmental activism self-identity results in environmental activism [76]. However, other types of pro-environmental actions cannot be explained with these specific self-identities. Recent studies suggest that individuals might have a more general kind of environmental self-identity that is associated with a broad range of pro-environmental preferences, intentions, and behavior [22,77]. A more general environmental self-identity can influence a wide range of pro-environmental behaviors. Therefore, targeting this general environmental self-identity might be a more viable tool in promoting pro-environmental actions. Hence in this study, we consider a general environmental self-identity to determine consumer organic food behavioral intention.

#### The Mediating Effect of Environmental Self-Identity

In the current study, we propose that consumption values have a positive relationship with consumers’ behavioral intention to consume organic products, and consumers’ environmental self-identity significantly mediates this positive relationship. 

The values which consumers derive from organic food consumption not only act as a motivation to consume the product, but also fulfill individuals’ environmental self-identity to be a pro-environment person. For example, functional value derived from organic food product provides not only functional benefits such as better quality, health, and economic benefits, but also influence individual’s environmental self-identity by providing environmental benefits such as ozone-friendly nature, and no use of chemical and pesticides which is harmful to the environment. Similarly, the social value associated with an organic food product raises an individual’s social image and status on the one hand and fulfills his environmental self-identity by contributing to the environment with clean production of organic food product on the other hand. Likewise, the conditional value associated with an organic product provides consumers with situational benefits in the form of nearness, subsidies and discounts and, at the same time, influences their environmental self-identity to perform pro-environmentally under worsening environmental conditions. Epistemic value derived from the consumption of organic product not only fulfills knowledge needs regarding product attributes but also provides information about individuals’ contribution to environmental protection. Building on similar logic, recent literature acknowledges that consumer self-identities mediate the relationship between consumer motivations/values and their organic food behavior [25,26,27]. More specifically, Van der Werff, Steg and Keizer [49] found that environmental self-identity mediates the link between consumer biospheric values and pro-environmental intentions and behavior. Based on the above discussion, we propose the following hypothesis:

**H6 a–f:**  Environmental self-identity mediates the effect of: (a) function value price. (b) function value quality. (c) social, (d) conditional, (e) epistemic, and (f) emotional value on behavioral intention to consume organic products.

### 2.5. Control Variables

We include health consciousness, gender, age, education and income level of respondents as control variables in the proposed model. These variables are likely to be associated with endogenous constructs of environmental self-identity and behavioral intention to consume organic food [78].

Health consciousness is defined as an individual’s readiness to comply with health actions [79]. Willingness to undertake health actions comprises of three components [80]: motivation to remain healthy, foreseen health risks postured by sickness and the chances that health-related action will lessen the health risks. A high health-conscious consumer considers organic food products healthier, and with better taste and quality and exhibits a more favorable attitude towards organic food consumption [37,81]. 

Previous studies also suggested that females are more conscious of environmental protection, and are more likely to consume organic product than males [5,82]. Also, young consumers are ready to change their consumption habits due to environmental concern [83] and have more positive intentions toward organic food consumption than older one [56,84]. Consumer educational and income level also significantly influence their organic food behavior. As a consumer with higher education and income level are more likely to consume organic food product [85,86]. 

## 3. Methodology

### 3.1. Procedure and Participants

**Figure 2:**
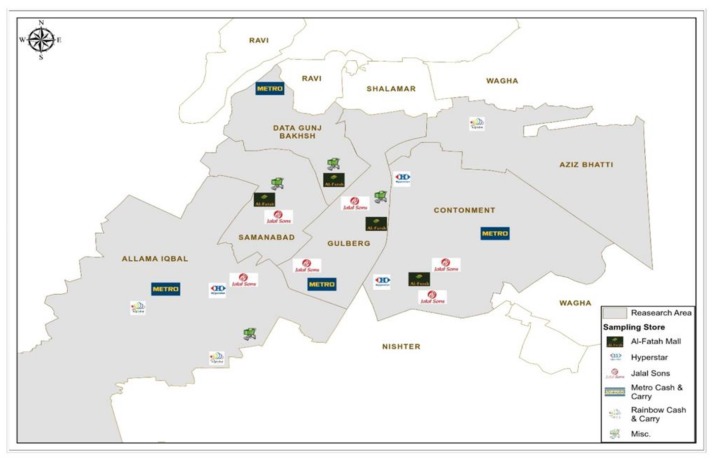
Distribution of samples.

In applying a quantitative approach to examine the proposed hypotheses, empirical data has been collected through a structured self-administered questionnaire. The sample questionnaires were distributed outside of 24 organic food outlets and departmental stores in different areas of Lahore (Pakistan) during September and October 2018, as shown in Figure 2. Lahore is the capital of Punjab Province and the second largest city of Pakistan, with an estimated population of more than 9.2 million. Informed consent was taken from respondents before participating in the study. The respondents were those with the experience of organic food consumption. By applying a random sampling method, consumers were approached who were coming out of the outlets and stores in the market. To ensure respondents have enough knowledge regarding organic food products, some primary questions were asked before surveying the respondents. A detailed questionnaire was given only to those individuals who had organic food awareness. A total of 500 survey questionnaires were distributed. Out of the questionnaires received back, 406 were fully complete, valid and usable for the study. This yielded an 81% response rate. Participation of respondents was entirely voluntary.

### 3.2. Demographic Profile of Respondents

The demographic profile of respondents is shown in Table 1. As to gender, 60.6% of the respondents were male. As to the age, 42.1% of the respondents aged between 25–35 years, followed by 18–24 years age group with 40.9% respondents. 36% of the respondents had a Master’s degree or above, and 30.5% were undergraduate. As to the occupation, 33.7% of the respondents were employees, and 30.5% were students. As to the income-level, about 33% of the respondents had a monthly income between PKRs 50,000 to PKRs 100,000 and 28.8% had a monthly income below 50,000 PKRs.

### 3.3. Development of Measurement Scale

The survey instrument used to collect data was consisted of three sections. The first section evaluated the consumer’s perception of organic food based on consumption values. The measurement items of consumption values including functional value price and quality, social, conditional, epistemic, and emotional value were adapted from Lin and Huang [8] and Biswas and Roy [34]. All constructs consist of 3 items, to measure it. The second section consisted of questions measuring consumers’ environmental self-identity with four items adapted from Van der Werff, Steg and Keizer [74] and Walton and Jones [87], and health consciousness with three items adapted from Singh and Verma [3] and Michaelidou and Hassan [81]. The third section measured consumer behavioral intention to purchase organic food with four items and was adapted from the scales of Singh and Verma [3] and Biswas and Roy [6]. Ranging from strongly disagree (1) to strongly agree (5), a 5-point Likert scale was used to measure all items in the above sections. The last part of the survey consisted of a respondent’s demographic information. All items used in the questionnaire are presented in Table 2. 

### 3.4. Common Method Variance

Since this study leads us to collect data from the same respondents for both independent and dependent variables, there are chances of common method variance existence in this study. We opted a methodological test named Harman’s one-factor test to measure the common method variance [88,89]. SPSS 24 is used to insert all items in principle components factor analysis. Outcome generated showed nine extracted factors with eigenvalues greater than 1.0, including one control variable. The total variance accounted for by this extracted factor was 73.69, while the first factor accounted for only 22.2 % of the variance. As principal component factor analysis doesn’t produce a single factor and also the first factor did not explain the majority of the variance, common method variance might not be an issue for this study.

### 3.5. Data Analysis

Statistical package for the social sciences (SPSS 24.0, IBM, Armonk, NY, USA) [90] was used to conduct a descriptive analysis. However, to test the proposed model, partial least square-structural equation modeling (PLS-SEM) technique was employed via SmartPLS 3.2.4 [91]. PLS-SEM is a wide-ranging multivariate technique to statistically examine the complex multivariable relationships in both measurement and structural models. It can handle complex models with a large number of latent variables even with three or lesser items. Also, it is more appropriate to use this technique for emerging areas of research with prediction oriented studies where the relationships between certain constructs have not been much explored yet [92,93,94]. 

## 4. Results

### 4.1. Partial Least Square Structural Equation Modeling

A two-stage approach of PLS-SEM was applied for conceptual model assessment, including the measurement model and the structural model. 

#### 4.1.1. Measurement Model Assessment

Initially, the measurement model was assessed through convergent and discriminant validity. Nine constructs were employed in this research. To evaluate convergent validity, values of factor loadings, average variance extracted (AVE) and composite reliability (CR) were measured [95] as shown in Table 2. All values of CR and AVE fall in the range of 0.835 to 0.869 and 0.560 to 0.688, respectively, exceeding the threshold values of 0.7 and 0.5 [95].

Factor loading of each item ranged from 0.720 to 0.862 which exceeds the cut-off value of 0.7. However, one item BI4 have the value of 0.644 which is acceptable, as values of composite reliability and average variance extracted are above threshold values [95].

To examine discriminant validity (DV), Fornell and Larcker [96] criteria and heterotrait-monotrait (HTMT) ratio were employed. The results of both statistical methods are shown in Table 3. To evaluate Fornell and Larcker [96] criteria square root of AVE of each construct was placed at diagonal, which exceeds its highest correlation coefficients with other constructs, signifying the fulfillment of discriminant validity. Similarly, HTMT ratio was evaluated by HTMT.90 criteria [97]. Table 3 showed that HTMT values of all the constructs were less than cutoff value of 0.90, accomplishing HTMT.90 criteria. Hence the results of discriminant validity for each construct are satisfactory.

#### 4.1.2. Structural Model Assessment

For structural model assessment, values and dimensions of beta coefficients and relevant t-statistics along with the measure of R^2^ for endogenous constructs are taken into consideration [95]. Bootstrapping method, based on 5000 samples was applied to measure path coefficients and their relative significance. Along with these standard calculations, we also measure the effect sizes (*f^2^*) for each structural path as recommended by Hair, Ringle and Sarstedt [95] in structural model assessment.

The calculated R^2^ values for both endogenous variables, environmental self-identity and behavioral intention to consume organic food, are 0.334 and 0.444, respectively. In Table 4, the values of beta coefficients, significance values and effect sizes f^2^ of each structural path are shown. Results reveal that functional value quality (β = 0.169, *p* ≤ 0.01), conditional value (β = 0.269, *p* ≤ 0.01), epistemic value (β = 0.125, *p* ≤ 0.01) and emotional value (β = 0.24, *p* ≤ 0.01) have significant positive impact on behavioral intention to consume organic food. Among these results, the conditional value has the strongest impact on behavioral intention to consume the organic product, followed by the emotional value. Hence our hypotheses H1b, H3, H4, and H5 are supported. However, functional value price (β = 0.021, *p* ≥ 0.05) and social value (β = 0.072, *p* ≥ 0.05) enter insignificant with behavioral intention to consume the organic product; these results do not support hypotheses H1a and H3.

Cohen [98] criteria were used to evaluate the measurement of the effect sizes, like 0.02, 0.15 and 0.35 for small, medium and large effects, respectively. Functional value quality, conditional value, epistemic value and the emotional value exceeded threshold criteria of 0.02 showing the small to medium effect as reported in Table 4. However, the functional value price and the social value showed no substantive significance. 

For control variables, the health consciousness has a significant positive effect on both environmental self-identity (β = 0.255, *p* ≤ 0.01) and consumers’ behavioral intention to consume organic food (β = 0.246, *p* ≤ 0.01). However, gender, age, education, and income-level all have insignificant influence on environmental self-identity and behavioral intention to consume organic food. 

#### 4.1.3. Mediation Effect of Environmental Self-Identity among Consumption Values and the Behavioral Intention to Purchase Organic Food

Our conceptual model posited that environmental self-identity will mediate the structural link between consumption values and consumer’s behavioral intention to consume organic food (i.e., hypotheses H6a to H6f). To examine mediation, this study followed the bootstrapping procedure, based on 5000 samples [95]. Specific indirect effect function introduced in Smart-PLS version 3.2.4 (SmartPLS GmbH, Bönningstedt, Germany) [91] was employed to report the result of the particular mediation path as shown in Table 5. Other than *p* values, 95% bias-corrected confidence intervals are also reported to confirm the significance of hypothesized indirect results, where the presence of zero value between the intervals will show the non-significant results [99].

Results in Table 5 reveal that the environmental self-identity significantly mediates: the relationship between the functional value quality and the behavioral intention to consume organic food (β = 0.031, *p* ≤ 0.01), the relationship between conditional value and the behavioral intention to consume organic food (β = 0.022, *p* ≤ 0.05), the relationship between epistemic value and the behavioral intention to consume organic food (β = 0.032, *p* ≤ 0.01) and the relationship between emotional value and the behavioral intention to consume organic food (β = 0.024, *p* ≤ 0.05). These results support the hypothesis H6b, H6d, H6e, and H6f. However, the environmental self-identity does not significantly mediate: the relationship between functional value price and the behavioral intention to consume organic food (β = −0.007, *p* ≥ 0.05) and the relationship between social value and the behavioral intention to consume organic product (β = −0.008, *p* ≥ 0.05). The last two results suggest that hypothesis H6a, H6c were not supported.

## 5. Discussion

This study has broadened our understanding of consumer behavior for organic food, by integrating the two lines of researches including the theory of consumption values and the environmental self-identity. Our findings in this study support the idea that targeting a general environmental self-identity of consumers is a promising approach to promote the consumption of the organic product in developing economies like Pakistan. Overall, our results suggest that environmental self-identity define the structural links between consumption values (i.e., function value quality, conditional, epistemic, emotional value) and the behavioral intention to consume organic food. 

First, by inspecting the direct impact of consumption values on consumers’ behavioral intention to consume organic food, we find that functional value quality positively affects the behavioral intention to consume organic food. However, the opposite result is observed for a functional value price. This supports hypothesis H1b and rejects H1a. This suggests that Pakistani consumers are more concerned about the quality of organic food, such as the healthy and nutritious ingredients, rather than the economic benefits attached to a lower price. This might be because recently the use of chemical fertilizers and pesticides in agriculture farming has caused adverse effects on people’s health. They are willing to pay the premium price for organic products to avoid the expenses that need to be incurred in case of bad health due to the consumption of low price non-organic food. Pakistan is a family-oriented society where the family head gives great importance to the health and well-being of family members instead of considering cost. These results are in line with the findings of Suki [60] who conclude that consumers pay more attention to the quality of organic products instead of price while making green purchase decisions. Some other studies have also suggested that green consumers are less sensitive to price [100,101] and are more willing to pay the premium price for organic food products [101,102]. Such willingness indicates consumers’ desire for a trade-off, where other factors of environmental and health concern outweigh the price factor in making product choice and purchase decisions. 

Our findings reject the H2 which states that social value attached to organic products increases consumers’ behavioral intention to consume organic food. This suggests that consumers do not feel a sense of social recognition or improvement in their social image while consuming organic food. Pakistan is an agriculture-based economy, and despite being migration from villages to cities, still, 60% of the population belongs to rural areas. Even people living in cities have strong connections and roots in villages. Most of the people have access to organic food products, and they treat it as a general commodity rather than considering it a product which improves their social image. Past studies also suggested that personal beliefs and norms are a more significant predictor of behavior than social concern [27,101]. These results are similar to the outcomes of Biswas and Roy [6], who did not find any significant influence of social value on sustainable consumption behavior.

We find that the conditional value has the strongest positive impact on the behavioral intention to consume organic food. This might be because, in recent years, extreme environmental situations had adversely affected individuals’ health and economic status in the country. Today consumers’ are more conscious of their consumption habits as compared to the past. This result has important implications for marketers and policymakers as it suggests that the easy availability and promotional activities regarding organic products are useful ways to promote organic food consumption. Consumers get convinced and motivated to consume organic products by such acts as they perceive them as an act of care and commitment towards masses by the concerned stakeholders. This result is the same as the results of Biswas and Roy [34] and Gonçalves, Lourenço and Silva [23] who found the significant positive influence of conditional value on consumers’ sustainable behavior.

Our results also reveal that epistemic value has a significant positive impact on the behavioral intention to consume organic food. Organic food companies in Pakistan use social media platforms to create awareness among people regarding the benefits of organic product consumption. This has increased consumers’ knowledge about organic food. Consumers tend to develop a positive perception of organic products as their knowledge of organic product attributes increases. Consumer’s desire for knowledge seeking is fulfilled by the availability of green tags and eco-labels on organic products, which help them to have a positive perception of the organic food product. Adding details to product tags and labels makes consumers’ decision easier and help them to recognize their role in preserving the environment. These results are consistent with the studies conducted by Lin and Huang [8] and Han, Wang, Zhao and Li [54]. 

Similarly, the significant positive impact of emotional value on behavioral intention to consume organic food suggests that organic food consumption evokes individuals’ positive emotions to perform in an environmentally responsible manner. Emotional association with the natural environment also facilitates to develop ecological consciousness among individuals that eventually impacts their purchase decisions of green product. These results are consistent with Gonçalves, Lourenço and Silva [23] who found a significant positive impact of emotional value on consumers’ green product choice decisions, but are different from Han, Wang, Zhao and Li [54] who reported an insignificant impact of emotional value on consumers’ intention to adopt green products. 

Our results suggest that among different control variables health consciousness found to have a significant positive impact on environmental self-identity and behavior intention to consume organic food. It depicts the acceptance of our premise that people in Pakistan are getting highly concerned about the health of their own and their family members. During the last few years, health issues caused by environmental problems have created havoc in society and not only hundreds of people lost their lives but also families badly hurt economically. Now irrespective of age, sex, educational and income levels a vast majority of people wants to contribute to green initiatives for themselves and for the society at large as well. These results are in similar to past research, stating that people health conscious and their concern for the environment are a key influence for sustainable and healthy food consumption [24,103].

Lastly, we examined whether environmental self-identity mediates the structural relationship between consumption values and the behavioral intention to consume organic food. To best of our knowledge, this study is the first to examine this mediation role of environmental self-identity between consumption values and the behavioral intention to consume organic food. We find that consumers’ environmental self-identity significantly mediates the relationship between some consumption values (i.e., function value quality, conditional value, epistemic value, and emotional value) and the behavioral intention to consume organic food. This suggests that the specific attributes of organic products, such as the bio-degradability and ozone-friendly aerosols, influence environmental self-identity of Pakistani organic food consumers which lead to pro-environmental behavior. Similarly, individual emotional attachment with the natural environment along with situational factors such as threatening environmental conditions and their knowledge regarding environmental problems influence their environmental self-identity. This indicates that eminence of conditional value, emotional value, epistemic value, and function value quality are instrumental in alluring consumer environmental self-identity, which lead to pro-environmental behavior intention. 

However, we do not find the mediating effect of environmental self-identity on the relationship between some consumption values (i.e., functional value price and social value) and the behavioral intention to consume organic food. This suggests that economic benefits associated with organic product do not influence an individual’s environmental self-identity in the Pakistani context. And individuals with higher environmental self-identity are willing to pay a premium price for organic product to act in an environmentally friendly manner. Moreover, social pressure and the improvement in self-image do not let an individual act in an environmentally friendly way. Their pro-environmental actions are based on personal belief and norms rather than social pressures.

Overall our findings support the idea that pro-environmental behavior is related to the self-perception of an individual [44] and consumers can be motivated to consume an environment-friendly product by focusing on their environmental self-identity. Previous research finds that past experience affects an individual’s self-identity and one’s self-identity can easily be changed [104]. Therefore, making product experience better in term of providing function value quality, condition, epistemic and emotional value positively influence environmental self-identity which leads to pro-environmental behavior intentions. Hence, targeting consumers’ environmental self-identity can be an effective tool to promote sustainable consumption behavior. 

## 6. Conclusions

In this study, we examine whether environmental self-identity mediates the structural relationship between consumption values and the behavioral intention to consume organic food. Using survey data of Pakistani consumers, we support that consumers’ environmental self-identity significantly mediates the relationship between consumption values and the behavioral intention to consume organic food.

Our findings have an important theoretical and practical implication. From the theoretical perspective, this study extends the existing knowledge by integrating the consumption values theory and general environmental self-identity approach in studying the consumers’ organic products choice behavior. Our results suggested that emphasis on improving functional value quality, conditional, epistemic and emotional value of organic product lead to positively influence individual environmental self-identity and their organic food behavior intention. However, results also found, that consumers’ are less concern regarding economic benefit and social approval gain through the consumption of organic food. Previous studies, such as Lee, Levy and Yap [50], De Pelsmacker, Moons and Barvarossa [51], Van der Werff, Steg and Keizer [74], have found that self-identities mediate the relationship between consumer motivations and their pro-environmental behavior. Adding to these studies, we find that one’s environmental self-identity significantly mediates the relationship between consumption values and organic food purchase intention. 

Second, our findings suggest that effective marketing campaigns can be designed keeping in view the consumers’ environmental self-identity. As such advertisements should not only introduce products to consumers but also let them realize their role to act in an environmentally friendly manner. More specifically, as we find that conditional value, which is due to the nearby availability of the product or discounts offered, is the most significant factor to affect consumer behavioral intentions. Therefore, government bodies and business firms should also play a prominent role in promoting organic food consumption by subsidizing products and establishing more sale points to make easy availability of the organic product. This practice can be instrumental in enhancing the conditional value of consumer, which motivate the consumer to participate in green consuming activities.

Similarly, we also find that epistemic, emotional and functional value quality are significant in affecting the consumers’ intention to consume organic products. As epistemic value is due to the knowledge fulfilling ability of the product, efficient use of traditional and social media campaign that introduces and educates about organic products can enhance epistemic value. A substantial amount of information on the product in terms of a product association with the environment should be provided for which individual can realize their contribution to environmental protection by consuming that product. Likewise, consumers having high emotional value consider sustainable consumption as an act of environment protection. Generally, they have intentions of doing good not only for themselves but also for society as a whole while having sustainable consumption. Marketers and practitioners can promote their organic products with marketing campaigns that evoke individual emotions towards the environment not only for themselves but for their future generations. Lastly, consumers’ concern about organic product quality should also be emphasized. As our results suggest that consumers are quality conscious regarding organic food, therefore manufacturers should ensure the quality of organic food to provide higher functional value quality of organic product. Along with that green attributes of organic product such as ozone-friendly aerosols and environmentally friendly nature must be ensured throughout manufacturing and distribution. 

### Limitations and Future Research

In this research, we use behavioral intentions to consume organic food. Future research can be done on actual behavior, and a comparison of intentions and actual behavior can also be performed to broaden our understanding of how much intentions, in reality, converts to actual behavior. As functional value price and social value proved non-significant in this study, future studies can be done to explore these values in detail for a clear understanding of consumer behavior. Also, the inclusion of certain moderating constructs such as consumers’ environmental value, product knowledge, and specific demographic details can also be explored. This study considered organic food to examine the consumer choice of pro-environmental behavior. Future research may contemplate other green products including high priced and energy efficient products and assess the proposed model relationship to determine consumer choice behavior in developing economies. Finally, this research was limited to a sample drawn from organic food consumers from the city of Lahore, Pakistan. To understand the breadth and depth of the organic food consumer intentions in Pakistan getting a sample from other provinces and cities including smaller cities will be instrumental.

## Figures and Tables

**Table 1:** Demographic profile of participants.

Constructs	Category	Frequency	Percentage
Gender			
	Male	246	60.6
	Female	160	39.4
Age (years)			
	18–24	166	40.9
	25–35	171	42.1
	36–45	55	13.5
	Above 45	14	3.45
Education			
	High School or less	26	6.5
	Diploma holders	40	10.0
	Intermediate	69	17.0
	Undergraduate	124	30.5
	Master or Above	145	36.0
Occupation			
	Student	136	33.5
	Government employee	62	15.3
	Company employee	137	33.7
	Business owner	20	4.9
	Housewife	20	4.9
	Professionals	31	7.6
Income (PKRs = Pakistani rupees)	<PKRs 50,000	117	28.8
	PKRs 50,001–PKRs 100,000	134	33.0
	PKRs 100,001–PKRs 150,000	77	19.0
	PKRs 150,001–PKRs 200,000	50	12.3
	PKRs 200,001–PKRs 250,000	19	4.7
	> PKRs 250,000	9	2.2

**Table 2:** Reliability and validity for constructs.

Constructs	Statements	Loadings	(CR)	(AVE)
Function value price				
	The organic food product is reasonably priced.	0.757	0.849	0.653
	The organic food product offer value for money.	0.835		
	The organic food product has a good economic value.	0.828		
Function value quality				
	The organic food product has expectable standard quality.	0.835	0.868	0.687
	The organic food product is made from non-hazardous substances.	0.835		
	Taste of organic food is good.	0.817		
Social Value				
	Purchase of organic food product will help me gain social approval.	0.760	0.847	0.649
	Purchase of organic food product will make a positive impression on other people.	0.862		
	Purchase of organic food product will improve the way I am perceived	0.790		
Conditional Value				
	I would buy organic food products if they were easy to acquire (accessible nearby).	0.832	0.869	0.688
	I would buy the organic food product instead of conventional products under worsening environmental conditions.	0.817		
	I will purchase organic food products over conventional substitutes if they are offered at a subsidized rate.	0.839		
Epistemic Value				
	I prefer to check the eco-labels and certifications on organic food products before purchase.	0.830	0.840	0.636
	I would prefer to gain substantial information on organic food products before purchase.	0.814		
	I am willing to seek out novel information.	0.746		
Emotional Value				
	Buying the organic food product instead of conventional products would feel like making a good personal contribution to something better.	0.799	0.839	0.636
	Buying the organic food product instead of conventional products would feel like the morally right thing.	0.839		
	Buying the organic food product instead of conventional products would make me feel like a better person.	0.750		
Environmental Self-identity				
	Acting environmental friendly is an important part of who I am.	0.778	0.863	0.612
	I am the type of person who acts environmental friendly.	0.799		
	I see myself as an environmental-friendly person.	0.827		
	I make significant changes in my lifestyle for environmental reasons	0.720		
Behavioral Intention				
	Given a choice between two substitute products, I intend to choose an organic food product in the future	0.804	0.835	0.560
	I am always interested in buying more organic food products for the family’s needs.	0.806		
	I will make a special effort to consume organic food product.	0.729		
	Given that organic food products are readily available, I predict that I would use them in the future.	0.644		
Health consciousness				
	I am very self-conscious about my health.	0.853	0.851	0.656
	I am alert to changes in my health.	0.836		
	I consume organic food as it considers good for health.	0.737		

Notes: AVE: average variance extracted, CR: composite reliability.

**Table 3:** Discriminant Validity.

Fornell-Larcker’s (1981) Criterion									
	BI	CV	EMO	EPV	ESI	FVP	FVQ	HC	SV
Behavioral Intention	0.749								
Conditional value	0.475	0.829							
Emotional value	0.471	0.403	0.797						
Epistemic value	0.334	0.291	0.362	0.798					
Environmental self-identity	0.415	0.357	0.371	0.364	0.782				
Functional value price	0.169	0.111	0.175	0.063	0.095	0.808			
Functional value quality	0.375	0.323	0.277	0.187	0.346	0.338	0.829		
Health consciousness	0.443	0.275	0.197	0.135	0.389	0.175	0.297	0.810	
Social value	0.330	0.274	0.401	0.196	0.264	0.245	0.348	0.296	0.805
Heterotrait-Monotrait Ratio (HTMT)									
Behavioral Intention									
Conditional value	0.622								
Emotional value	0.636	0.533							
Epistemic value	0.462	0.394	0.498						
Environmental self-identity	0.545	0.454	0.486	0.480					
Functional value price	0.224	0.140	0.240	0.103	0.121				
Functional value quality	0.481	0.420	0.374	0.250	0.442	0.438			
Health consciousness	0.595	0.365	0.275	0.188	0.509	0.238	0.388		
Social value	0.442	0.362	0.549	0.255	0.337	0.343	0.456	0.413	

FVP = functional value price, FVQ = functional value quality, SV = social value, CV = conditional value, EPV = epistemic value, EMO = emotional value, ESI = Environmental Self-identity, HC = Health consciousness, BI = behavioral intention to purchase organic food.

**Table 4:** Structural estimates (Hypotheses testing).

Hypothesized Path	*β*-Value	t-Value	*f^2^*	Results
H1a: Functional value price—Behavioral intention	0.021	0.486	0.001	Not supported
H1b: Functional value quality—Behavioral intention	0.169	3.286 ^**^	0.036	Supported
H2: Social value—Behavioral intention	0.072	1.42	0.006	Not supported
H3: Conditional value—Behavioral intention	0.269	5.443 ^**^	0.089	Supported
H4: Epistemic Value—Behavioral intention	0.125	2.420 ^**^	0.021	Supported
H5: Emotional value—Behavioral intention	0.240	5.082 ^**^	0.064	Supported

Notes: ** *p* ≤ 0.01.

**Table 5:** Specific indirect effect.

Hypothesized Path	β-Value	t-Value	Confidence Interval	Status
**H6a**: FVP → ESI → BI	−0.007	0.853	[−0.023; 0.005] N-sig	Not supported
**H6b**: FVQ → ESI → BI	0.031	2.266 **	[0.010; 0.055] Sig	Supported
**H6c**: SV → ESI → BI	0.008	0.871	[−0.003; 0.027] N-sig	Not supported
**H6d**: CV → ESI → BI	0.022	1.635 *	[0.004; 0.049] Sig	Supported
**H6e**: EPV → ESI → BI	0.032	2.241 **	[0.011; 0.057] Sig	Supported
**H6f**: EMO → ESI → BI	0.024	1.680 *	[0.005; 0.051] Sig	Supported

Note: * *p* ≤ 0.05; ** *p* ≤ 0.01, FVP = functional value price, FVQ = functional value quality, SV = social value, CV = conditional value, EPV = epistemic value, EMO = emotional value, ESI = Environmental Self-identity, BI = behavioral intention to purchase organic food.
